# Artemisinin analogue SM934 attenuate collagen-induced arthritis by suppressing T follicular helper cells and T helper 17 cells

**DOI:** 10.1038/srep38115

**Published:** 2016-11-29

**Authors:** Ze-Min Lin, Xiao-Qian Yang, Feng-Hua Zhu, Shi-Jun He, Wei Tang, Jian-Ping Zuo

**Affiliations:** 1 Laboratory of Immunology and Virology, Shanghai University of Traditional Chinese Medicine, Shanghai 201203, People’s Republic of China; 2 Laboratory of Immunopharmacology, Shanghai Institute of Materia Medica, Chinese Academy of Sciences, Shanghai 201203, People’s Republic of China

## Abstract

SM934 is an artemisinin analogue with immunosuppressive properties and potent therapeutic activity against lupus-like diseases in autoimmune mice. In this report, the therapeutic efficacy and underlying mechanisms of SM934 on rheumatoid arthritis (RA) was investigated using collagen-induced arthritis (CIA) in DBA/1J mice. We demonstrated that SM934 treatment alleviate the severity of arthritis in CIA mice with established manifestations. The therapeutic benefits were associated with ameliorated joint swelling and reduced extent of bone erosion and destruction. Further, administration of SM934 diminished the development of T follicular helper (Tfh) cells and Th17 cells and suppressed the production of pathogenic antibodies, without altering the proportion of germinal center B cells. *Ex vivo*, SM934 treatment inhibited the bovine type II collagen (CII) induced proliferation and inflammatory cytokines secretion of CII -reactive T cells. *In vitro*, SM934 impeded the polarization of naïve CD4^+^ T cells into Tfh cells and the expression of its transcript factor Bcl-6. Moreover, SM934 decreased the IL-21-producing CD4^+^ T cells and dampened the IL-21 downstream signaling through STAT3. These finding offered the convincing evidence that artemisinin derivative might attenuate RA by simultaneously interfering with the generation of Tfh cells and Th17 cells as well as the subsequent antibody-mediated immune responses.

Artemisinin (Qinghaosu) is a sesquiterpene lactone bearing a peroxy isolated from *Artemisia annua L*^1^,^2^. Artemisinin and its derivatives have been widely used in the world as first-line antimalarial drug for decades. The interaction between artemisinin and Fe^2+^ leads to the cleavage of peroxide bond in artemisinin, which result in the release of C-centered free radicals, the potent alkylating species then directly impair functions of malarial mitochondria^3^,^4^,^5^. In addition to the outstanding antimalarial activity, artemisinin and its derivatives possess significant immunosuppressive effects both *in vitro* and *in vivo*^6^,^7^,^8^,^9^,^10^,^11^. We have previously reported that, among synthesized artemisinin derivatives with various structures, SM934, the novel water soluble artemisinin analogue exhibits promising therapeutic effect on multiple autoimmune diseases by suppressing the differentiation and expansion of pathologic T helper cells^12^,^13^,^14^ and accumulation of plasma cells^15^. Nevertheless, the impact of artemisinin analogues on T follicular helper (Tfh) cells, the critical participator in germinal center (GC) formation, remains largely unclear.

Germinal centers (GCs) are specialized structures that develop within B cell follicles of secondary lymphoid tissues where antigen-specific B cells undergo somatic hypermutation and affinity maturation, and then differentiate into antibody-producing plasma cells and memory B cells^16^,^17^,^18^.

As a recently identified CD4^+^ T cell subset, Tfh cells, have been found to be present in GCs, provide selection signals and developmental cues for the proliferation, differentiation and class switching of antigen-selected high-affinity GC B cells^19^,^20^,^21^,^22^. The phenotypic and functional features of Tfh cells include expression of the chemokine receptor CXCR5, costimulatory molecules such as inducible T cell costimulatory (ICOS), programmed death 1 (PD-1), as well as the master transcription factor B-cell CLL/lymphoma-6 (Bcl-6) and the cytokine interleukin-21 (IL-21)^19^.

T helper 17 (Th17) cells, are highly proinflammatory effector T cells that are characterized by the production of high amounts of IL-17, IL-21, and IL-22. Recent research indicated that Th17 cells have greater capacity to provide cognate B cell help than T helper 1 (Th1) cells, and support higher expansion of antigen specific B cells^23^. Moreover, IL-17 itself could drive B cells to undergo preferential isotype class switch recombination and favor spontaneous GC formation in mouse model of autoimmunity^24^,^25^.

IL-21 signaling is important for GC persistence and function, B cell isotype switching and response to protein antigen. Both of Tfh and Th17 cells are considered as the major source of IL-21, conversely, IL-21 facilitates Tfh and Th17 cells lineage commitment in an autocrine fashion^26^,^27^. Operating in the same way as IL-6, IL-21 signals through the signal transducer and activator of transcription 3 (STAT3), in promoting or sustaining Tfh and Th17 development^26^,^27^.

Rheumatoid arthritis (RA) is an autoimmune disease characterized by synovial inflammation, cartilage damage and bone destruction. Persistent presence of circulating autoantibodies and dysregulated lymphocyte activation have been indicated to be associated with the pathogenesis of RA^28^,^29^,^30^. Increased frequency of circulating Tfh cells accompanied by higher levels of serum IL-21 have been reported in patients with rheumatoid arthritis and systemic lupus erythematosus (SLE)^31^,^32^. Accumulated reports indicated that Tfh cells may account for RA pathogenesis by providing a fostering milieu for self-reactive B cells, and circulating Tfh cells may serve as a biomarker of the pathogenesis and for evaluating the therapeutic responses of RA patient^33^,^34^. The Th17 population, have been shown to play a key role in orchestrating inflammatory response, contribute to the extracellular matrix destruction and bone resorption in RA, by means of inducing IL-6 and matrix proteases in synoviocytes from RA patients^35^,^36^.

Here we report the effect of SM934 on the collagen-induced arthritis (CIA) in DBA/1 mice, the widely used experimental model to investigate the pathogenesis and therapeutic agents of RA. We found that SM934 ameliorated the severity of arthritis in CIA mice via inhibiting development of Tfh and Th17 cells as well as autoantibodies production. Additionally, we demonstrated that SM934 could prohibit the IL-21-mediated signaling pathway by preventing the activation of STAT3.

## Results

### SM934 prevented arthritis progression in CIA mice

**Figure 1:**
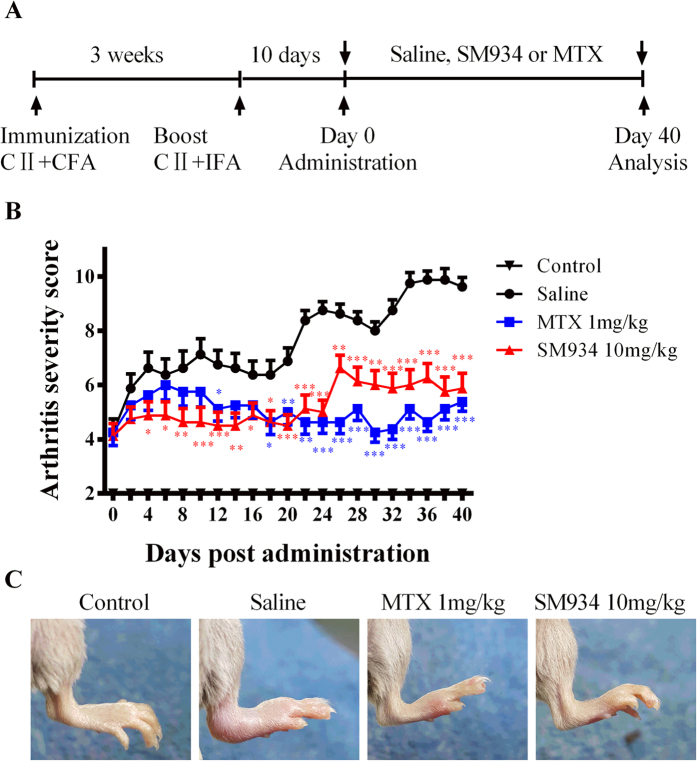
Effects of SM934 on disease progression in CIA mice. (**A**) Timetable of CIA induction and treatment strategy in CII-immunized DBA/1J mice. All mice were sacrificed on day 40 post administration. (**B**) Arthritis severity scores of CIA development in CIA mice were recorded every two days after administration. Values are the mean ± SEM (n = 8). *P < 0.05, **P < 0.01 and ***P < 0.001 compared with the saline-treated CIA mice (saline). (**C**) Representative photographs of showing the gross features of left hind paws at day 14.

To investigate the effect of SM934 on arthritis, the CIA model in DBA/1J mice was used. CIA mice with established rheumatoid arthritis manifestations such as erythema and edema in joints were orally administrated with saline, MTX or SM934 for 40 days (Fig. 1A). Overall arthritis severity in CIA mice was assessed by macroscopic clinical scoring. Administration of SM934 (10 mg/kg) significantly attenuated the clinical signs of arthritis since 4 days after treatment, indicated by the reduction in the mean arthritis score, the therapeutic effect was comparable with the methotrexate (MTX, 1 mg/kg), a disease-modifying antirheumatic drug (DMARD) treatment control used in comparison with arthritic group (Fig. 1B). Representative photographs displaying morphological changes of left hind paws of mice from individual group on day 14 after treatment are shown in Fig. 1C, macroscopic evidence of arthritis such as erythema or swelling was markedly observed in saline-treated CIA mice compared with normal DBA/1J mice, while SM934 and MTX treatment significantly ameliorated arthritis severity in CIA mice.

### SM934 reverted bone destruction in CIA mice

**Figure 2:**
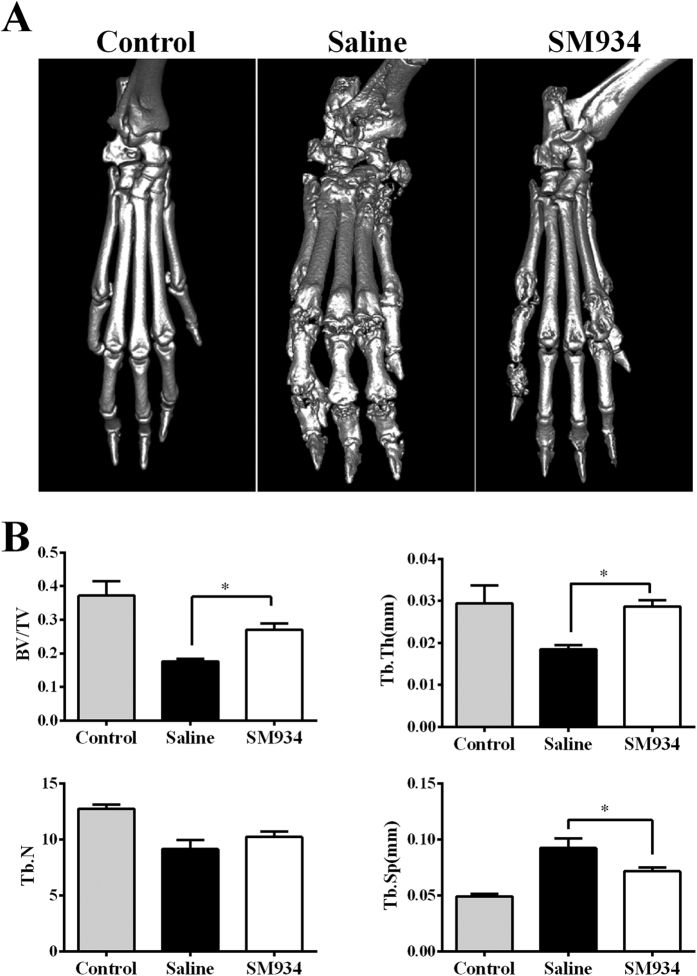
Micro-CT analysis confirmed the efficacy of SM934 in CIA mice. (**A**) Representative three-dimensional reconstruction of right hind paw of normal control mice and CIA mice treated with saline or SM934, respectively. (**B**) Histomorphometric analysis of the distal tibia for each treatment group, showing the values of four parameters including bone volume/total volume (BV/TV), trabecular number (Tb. N.), trabecular thickness (Tb. Th.), and trabecular spacing (Tb. Sp.) of mice control mice and CIA mice treated with saline or SM934. Values are the mean ± SEM (n = 8). *P < 0.05 compared with the saline-treated CIA mice (saline).

Micro computed tomography (micro-CT) scan was performed to validate the efficiency of SM934 on bone erosion in CIA mice. Three-dimensional reconstruction of hind right paws by micro-CT demonstrated severe bone erosion within their periarticular bone of paws of the saline-treated CIA mice, in marked contrast to normal DBA/1J mice, as well as to SM934-treated mice (Fig. 2A). Moreover, as shown in Fig. 2B, saline-treated CIA mice showed a remarkable decrease of the bone volume/total volume (bone volume fraction, BV/TV), trabecular number (Tb. N.), trabecular thickness (Tb. Th.), and increase of the trabecular spacing (Tb. Sp.) compared with healthy controls. SM934 treatment significantly prevented these changes in BV/TV, Tb. Th. and Tb. Sp. Although the trabecular number was higher in SM934-treated group compared to the saline-treated group, there was no statistically significant difference.

### SM934 dampened T cell-dependent immune responses in CIA mice

**Figure 3:**
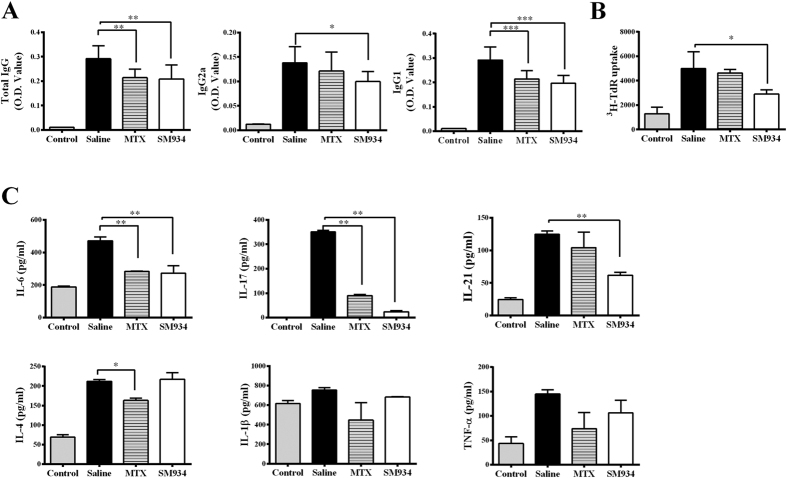
Effects of SM934 on CII-specific immune response in CIA mice. (**A**) Serum levels of total anti-CII IgG, IgG2a and IgG1 of CIA mice and normal control mice. Values are the mean ± SEM (n = 8). *P < 0.05, **P < 0.01 and ***P < 0.001 compared with the saline-treated CIA mice (saline). (**B**) B cell-depleted splenocytes isolated from CIA mice were stimulated with CII (10 μg/ml) and cultured for 72 h. Cell proliferation was measured by the [^3^H]-thymidine incorporation assay. (**C**) Supernatants were collected for cytokines detection by ELISA. All data are showed as mean ± SEM. *p < 0.05, **p < 0.01 compared with the cell culture of saline-treated CIA mice (saline). Results are derived from 2 independent experiments with similar pattern in each treated group (n = 8 in each group).

The humoral response against collagen-specific autoantigen is essential for CIA pathogenesis^37^,^38^, the effect of SM934 on serum antibodies (Abs) production was assessed at the end of treatment. Serum Ab levels from individual mouse were examined using isotype-specific ELISAs. The serum levels of anti-CII total IgG, IgG1 and IgG2a antibody decreased significantly after SM934 treatment, compare with saline-treated mice (Fig. 3A). Moreover, we used mice immunized with sheep red blood cells (SRBCs) and ovalbumin (OVA) to confirm the impact of SM934 on the robust antibody response. SM934 treatment significantly lower serum level of SRBC-specific and OVA-specific antibody respectively, compared with saline-treated control mice (Supplementary Fig. S1).

Since the autoreactive T cells plays a pivotal role in the pathogenesis of CIA mice, CII-induced T cell proliferation was conducted using B cell-depleted splenocytes from different groups. CII-stimulated B cell-depleted splenocytes obtained from CIA mice treated with SM934 exhibited less proliferation in comparison with the cells from saline-treated ones (Fig. 3B). The levels of inflammatory mediators were measured in supernatants of CII or medium cultured B cell-depleted splenocytes. Results indicated that the amounts of IL-6, IL-17, IL-21, IL-4 and TNF-α markedly elevated in the cell cultures of saline-treated CIA mice. SM934 treatment lowered the amount of IL-6, IL-17 and IL-21 in cultured cells responded to CII stimulation, while the concentration of IL-1β, IL-4 and TNF-α did not changed much (Fig. 3C). Taken together, the suppressive effects on CII-specific antibodies production and T cell response, suggesting that SM934 might participate in a T cell-dependent immune response in CIA mice.

### SM934 suppressed the Tfh and Th17 cells development in CIA mice

**Figure 4:**
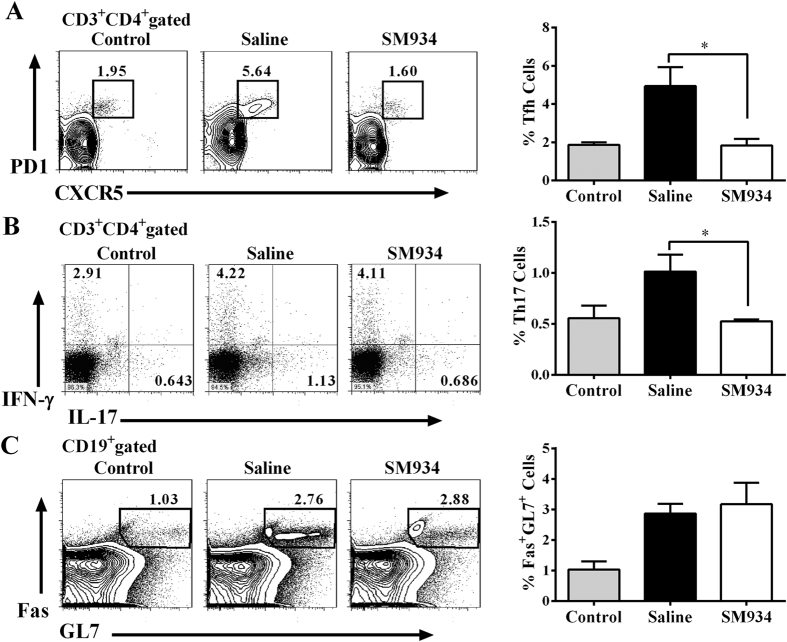
SM934 restrained Tfh and Th17 cells development but not GC B cells in CIA mice. Flow cytometric analysis of Tfh cells by PD-1 and CXCR5 staining (**A**), Th17 and Th1 cells by IL-17 and IFN-γ staining (**B**), or GC B cells by Fas and GL7 staining (**C**) in splenocytes from CIA mice. The representative (left) and statistical results (right) were showed. Values are the mean ± SEM. *p < 0.05 compared with the saline-treated CIA mice (saline). Results are derived from 2 independent experiments with similar pattern in each treated group (n = 8 in each group).

Flow cytometric analysis demonstrated that CD4^+^ CXCR5^+^ PD-1^high^ Tfh subset and CD4^+^ IL-17^+^ Th17 subset augmented remarkably in splenocytes from saline-treated CIA mice compared with normal control mice, and SM934 treatment largely abrogated the expansion of the Tfh and Th17 population (Fig. 4A,B). On the contrast, neither proportion of FAS^+^ GL-7^+^ GC B cells (Fig. 4C) nor the frequency of Th1 (statistical result in Supplementary Fig. S2) or regulatory T (Treg) cells (Supplementary Fig. S2) changed notably after SM934 treatment. In order to further confirm the effect of SM934 on Tfh and Th17 cells *in vivo*, we introduced the SRBC- and OVA-immunized mice treated with SM934 for 9 day and 7 day respectively. As expected, in both experiments, SM934 treatment significantly diminished Tfh population in comparison with saline-treated ones. SM934 treatment also significantly suppressed the Th17 expansion in OVA-immunized mice, whereas no statistical significance was observed between SRBC-immunized mice treated with SM934 and with saline (Supplementary Fig. S3).

### SM934 repressed Tfh cells differentiation *in vitro*

**Figure 5:**
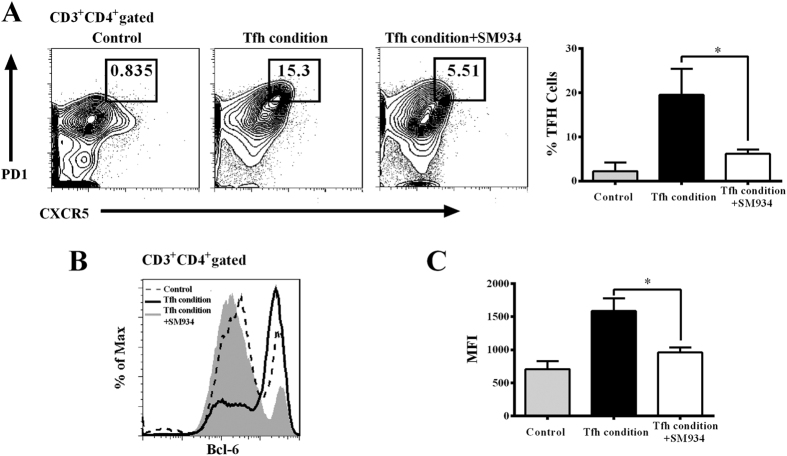
SM934 suppressed Tfh cells differentiation in culture. (**A**) Representative (left) and statistical (right) flow cytometric results of the Tfh cells differentiation. Naïve T cells were cultured under Tfh polarization condition, in the absence or presence of SM934 (10 μM) for 4 days. (**B**) Flow cytometric analysis of intracellular transcription factor Bcl-6. Shaded area depicted Th0 cells, full line represented Tfh cell culture, and dotted line showed Tfh cell cultured with DZ2002. (**C**) Bars showed mean fluorescence intensity (MFI) of Bcl-6 expression. Values are the mean ± SEM (n = 3). *p < 0.05 compared with the cell culture under Tfh differentiation condition. Results are derived from 3 independent experiments with similar results.

To further determine whether SM934 exerted an direct impact on the development of T helper cells, naïve CD4^+^ T cells from splenocytes of C57BL/6 mice were induced towards to Tfh cell differentiation in the presence or absence of SM934 for 4 days, using the naïve CD4^+^ T cells cultured under neutral conditions as Th0 control. As we reported previously, SM934 (10 μM) could significant impede differentiation of Th1 and Th17 cells and exerted little influence on Treg polarization *in vitro*^13^, we thus sought to identify the impact of SM934 on the Tfh cells differentiation. As shown in Fig. 5A, the frequency of Tfh cells were significantly decreased when treated with SM934 (10 μM).

We simultaneously examined whether SM934 modulated the expression of Bcl-6, the unique transcript factor for Tfh programming. As shown in Fig. 5B, the cell culture under Tfh differentiation condition demonstrated a sharp increase of Bcl-6 expression, while SM934 treatment largely abrogated the intracellular expression of Bcl-6.

### SM934 repressed IL-21 production from T helper cells and disrupted the downstream signaling

Apart from costimulatory signals providing cognate help to GC B cells differentiation, IL-21, one of the primary cytokines derived from Tfh and Th17 cells, acts directly on the formation of plasma cells and the maintenance of GCs^39^.

**Figure 6:**
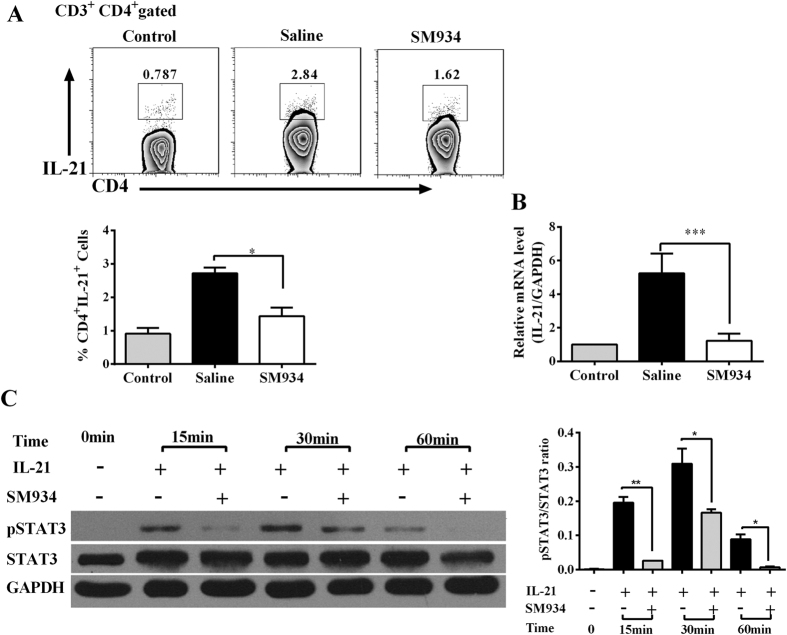
SM934 inhibited generation of IL-21-producing T helper cells and impeded downstream signaling transduction. (**A**) Representative (up) and statistical (low) results of the IL-21^+^ CD4^+^ T cell frequency in splenocytes from CIA mice determined by flow cytometry. (**B**) IL-21 mRNA level in splenocytes from CIA mice was measured by real-time PCR. Values are the mean ± SEM. Results are derived from 2 independent experiments with r pattern in each treated group (n = 8 in each group). (**C**) Western blot analysis of phosphorylated STAT-3 protein, in B cell-depleted splenoctys from C57BL/6 mice stimulated with exogenous IL-21 in the presence or absence of SM934 at indicated time points. Total STAT-3 and GAPDH proteins were used as the loading control. The full-length blots are included in the Supplementary Information 2. Representative blots from 3 experiments are showed in left panel. Right panel showed quantification of Western blotting images by ImageJ. Values are the mean ± SEM (n = 3). *p < 0.05, **p < 0.01 compared with the IL-21 treated cells in the absence of SM934. Results are derived from 3 independent experiments with similar results.

In CIA mice, we observed elevated level of IL-21 on the CD4^+^ T cells, in line with the up-regulated of IL-21 mRNA expression. SM934 treatment induced pronounced decrease of the percentage of CD4^+^ IL-21^+^ cells (Fig. 6A). Accordingly, real-time PCR analysis revealed that IL-21 expression in splenocytes of SM934-treated CIA mice decreased to approximately 23% of saline-treated ones (Fig. 6B).

We further investigated what effect of SM934 would have on the downstream signaling of IL-21 on T cells. B cell-depleted splenocytes from normal C57BL/6 mice stimulated with IL-21 were simultaneously cultured with or without SM934 (10 μM) for 0 to 60 minutes. We observed the SM934 treatment significantly inhibited the phosphorylation of STAT3 at 5, 30 and 60 minutes time points (Fig. 6C). Collectively, these results revealed that SM934 could abolish the IL-21 secretion and downregulate its downstream signaling through STAT3.

## Discussion

Recent years, artemisinin and its derivatives with immunosuppressive activities have been investigated as promising therapeutic candidates for multiple autoimmune disorders. In present study, we demonstrated that SM934 treatment ameliorated the severity of arthritis in CIA mice, in improving the clinical conditions and suppressing the production of pathogenic antibodies.

Collagen-induced arthritis is the most widely used animal model for the evaluation of novel therapeutic strategies for rheumatoid arthritis and shares both immunological and pathological features of human RA. There are considerable evidence implied that CII-reactive CD4^+^ T cells as the primary mediators of disease induction and complement-fixing anti-CII autoantibody production by B cells should be the major immune mechanism instigate the local inflammatory response resulting production of cytokines and inflammatory mediators^40^,^41^,^42^. Our results showed that SM934 treatment could prevent the aggravation of arthritis manifestation and suppress the anti-CII antibody production, especially for the IgG2a subclass, which dominated the anti-CII response in CIA. Further, SM934 inhibited the expansion of Tfh cells and Th17 cells, the potent supporters for germinal center reaction and generation of antigen specific B cells in autoimmune diseases. Intriguingly, SM934 treatment exerted little impact on the frequency of GC B cell, and did not affected the Th1 or Treg proportion either.

It is well documented that the GC B cells are precursors of antigen-specific memory B cell and long-lived plasma cells (PC). As for the role of Tfh cells, GC B cells and Th cell-B cell interaction in an antigen-specific response, Hu and colleagues^43^ suggested a reasonable illustration, using a SRBC-challenged mice and co-culture experiment, that it was the generation of Tfh cells, rather than direct activation B cells by Tfh cells, should be the primary mechanism modulating IgG antibody production. Moreover, there are plenty of molecular and cellular underpinnings in the development of B cell memory and PC formation, including molecular interactions that organize at cellular interfaces of Th cells and GC B cells. Reports have identified numbers of surface molecules implicated in the checkpoint of GC B cell development: CD40-CD40L and ligation play an essential role in class switch recombination and GC formation^44^; ICOS-B7RP-1(ICOSL) costimulation is critical for production of cytokines lead to B cell activation and differentiation^43^; CD30-CD153 interactions have an inhibitory effect antibody production *in vivo*^45^. Together, during the formation of antibody-producing cells, Tfh cells provide necessary help to GC B cells in terms of costimulatory signals and differentiation factors. In current study, SM934 impeded the propagation of Tfh cells and controlled autoantibody production in CIA mice without altering the population of GC B cells. We speculated that the suppression on the generation Tfh cells and IL-21, the critical executor of B cell fate, should be responsible for this phenomenon. Nevertheless, what specific action would SM934 have on memory B cells and long-live plasma cells in RA status, could it regulate the expression of surface molecules on Tfh cells were unknown and required further investigation.

Similar as our previous reports^12^,^13^, in the present study, SM934 retarded the development of Th17 cells both in the autoimmune animals and cellular culture *in vitro*. Accumulating reports have indicated a distinct role of Th17 cells in supporting B cell responses. Bauquet *et al.*^46^ demonstrated that Th17 cells and Tfh cells shared several features, including the expression of ICOS and IL-21. Patakas *et al.*^23^ stated that Th17 cells have a significant role earlier in the initiation/development of the GC responses, by using T cell and B cell receptor transgenic mice specific for model antigens. Mitsdoerffer *et al.*^24^ found that even IL-17 on its own, could drive class switch recombination in myelin oligodendrocyte glycoprotein (MOG)-immunized 2D2 TCR transgenic mice. As one of the major producer of IL-17 and IL-21, Th17 cells might not only act as proinflammatory characters^47^ but also incitement of excessive GC reaction and antibody production in RA pathology. In light of these properties, the subsequent effect of the suppression on Th17 cells by SM934, could facilitate the general repression of antigen-specific antibody production in CIA mice as well as in SRBC- and OVA-challenged mice.

IL-21 exerts pleiotropic actions on the immune system. Besides engagement in the function of T cells, natural killer (NK) and dendritic cells (DCs), IL-21 exerts profound influence on B cell biology. Our results showed that SM934 treatment inhibited the production of IL-21 from CD4^+^ T cells and abolished the downstream signaling of IL-21 through STAT3, coincided with the decreased level of antibodies and reduction of Tfh cells and Th17 cells. Actually, IL-21 serves as an autocrine factor secreted by Tfh and Th17 cells that promotes or sustains Tfh and Th17 lineage commitment in a STAT3-dependent manner^27^,^48^. Moreover, STAT3 not only plays as the transduction element of IL-21 signaling, but also binds the *Il21* promoter directly and modulates IL-21-responsive genes^49^. Therefore, SM934 treatment could interfere IL-21 circuit including IL-21 per se and STAT3 activation, which thus reinforce the inhibitory effects of SM934 on Tfh and Th17 cells. Furthermore, IL-21 can induce various fate on B cells, depending on the interplay with costimulatory signals and on the developmental stage of a B cell: in B cells that encounter antigen and receive T cell help, IL-21 induces survival, proliferation, isotype switching, and differentiation to antibody-secreting PCs; in those B cells receiving signals via BCR alone, as can be the case for some autoantigens, or via TLR, IL-21 costimulation causes apoptosis^50^. These features of IL-21 suggested that there might be a discrepancy between the mechanisms of SM934 on the spontaneous autoimmune diseases and antigen-induced immune responses, relevant to IL-21, which is worthy an attentive investigation in further studies.

## Materials and Methods

### Animal ethical statement

The animal experiment was carried out in strict accordance with the institutional ethical guidelines on animal care and were approved by the Institute Animal Care and Use Committee (IACUC) at the Shanghai Institute of Materia Medica, Chinese Academy of Sciences (IACUC protocol# 2015-12-ZJP-46 for DBA/1J mice, # 2015-01-ZJP-35 for C57BL/6 and BALB/c mice).

### Animals

Male DBA/1J, female C57BL/6 and BALB/c mice were purchased from Shanghai Laboratory Animal Center of the Chinese Academy of Sciences. All mice were housed in a pathogen-free facility and rabbits were housed in clean-grade animal cabin with free access to standard laboratory water and food, and kept in a 12 h light/dark cycle with controlled humidity (60–80%) and temperature (22 ± 1 °C).

### Collagen-induced arthritis

The male DBA/1J mice were randomly divided into 4 groups (n = 8 per group), 3 groups were immunized at the tail base with 100 μg bovine type II collagen (CII, Tokyo, Japan) in 0.1 M acetic acid emulsified equal volume complete Freund’s adjuvant (CFA) containing Mycobacterium tuberculosis strain H37Rv (Wako Pure Chemical Industries Ltd., Osaka, Japan). A boost injection of 100 μg collagen-incomplete Freund’s adjuvant (IFA) emulsion was given in the same manner 3 weeks later. From day 10 after booster immunization, immunized groups were orally administered with saline, MTX (1 mg/kg/day) or SM934 (10 mg/kg/day) for consecutive 40 days, while normal controls were administered with saline. The arthritis severity of mice was monitored every two days. At the end of treatment, all 4 groups of DBA/1J mice were sacrificed, and serum, hind paws and splenocytes were then collected.

### Clinical assessment of arthritis

The clinical severity of arthritis was scored as previously described^51^. Briefly, each limb was graded based on a scale of 0 to 4 according to the following scale: 0 = normal; 1 = detectable arthritis with erythema one or several digits; 2 = erythema and moderate swelling extending from the ankle to the midfoot; 3 = severe swelling and redness from joint to digit; and 4 = maximal swelling with ankyloses. The severity was described as the cumulative score of four limbs (the maximum score for each mouse is 16).

### Micro-CT scans and image analysis

Three-dimensional reconstruction of the hind knee and ankle joints were obtained by Micro-CT examination (Inveon MM system, Siemens Preclinical Solutions) at the end of treatment. Briefly, after the mice in different groups being killed using ether anesthesia, the hind limbs were removed and fixed in 4% paraformaldehyde. The samples were scanned with micro-CT, and images were acquired at an effective pixel size of 8.5 μm, voltage of 80 kV, current of 500 μA and exposure time of 1000 ms in each of the 360 rotational steps. Parameter were calculated using an Inveon Research Workplace (Siemens Medical Solutions) using manufacturer-supplied software as follows: bone volume/total volume (bone volume fraction, BV/TV), trabecular number (Tb. N.), trabecular thickness (Tb. Th.), and trabecular spacing (Tb. Sp.).

### SRBC-immunized BALB/c mice

Naïve female BALB/c mice were injected intraperitoneally with 2.5 × 10^8^ SRBCs. Saline or SM934 (10 mg/kg) were orally administered for consecutive 9 days after immunization. Five of the unimmunized female BALB/c mice were used as normal controls.

### OVA-immunized C57BL/6 mice

Naïve female C57BL/6 mice were immunized with OVA-CFA emulsion as described previously^52^, then were treated with saline or SM934 (10 mg/kg/day) for consecutive 7 days. Five of the unimmunized female C57BL/6 mice were used as normal controls.

### Flow cytometric analysis

Single-cell suspensions were prepared from spleens or from cell cultures. Antibodies for surface staining, PerCP-Cy5.5-conjugated anti-CD3, PE-conjugated anti-CD4, FITC-conjugated anti-PD-1, APC-conjugated anti-CXCR5, Alexa Fluor 647-conjugated anti-GL7 and FITC-conjugated anti-PD-1 were purchased from BD bioscience. For intracellular staining, splenocytes were first stained with surface markers followed by fixation and permeabilization using FoxP3 Staining Buffer set purchased from eBioscience^53^. Cells were labelled intracellularly PE-conjugated anti-Bcl-6 (eBioscience, San Diego, CA, USA), PE-conjugated anti-IL-17, APC- conjugated anti-IFN-γ or PerCP-Cy5.5- conjugated anti-FoxP3 (BD Biosciences, San Diego, CA, USA). Flow cytometry was performed on 4-laser/13-color BD LSRFortessa (BD Biosciences) and data were analyzed using FlowJo software (Tree Star, Ashland, OR).

### Antibody and cytokine assays

At the end of the animal experiments, serum were collected and levels of specific antibodies were measured by enzyme-linked immunosorbent assay (ELISA).

Serum titers of anti-CII or anti-SRBC IgG were determined as previously described^51^,^54^. Briefly, 96-well ELISA plate were coated with bovine CII (50 μg/ml) or extracted SRBC membranes (20 μg/ml) (prepared as described previously^55^) overnight at 4 °C. Each diluted serum sample was added and incubated. Horse-radish peroxidase (HRP) conjugated goat anti-mouse IgG (H + L), IgG1and IgG2a Abs (Invitrogen, San Diego, CA, USA) were used. The optical density is measured spectrophotometrically at 450 nm.

Cytokines in sera and culture supernatants were detected using mouse IL-6, IL-17A (BD Biosciences, San Diego, CA) and IL-21 (R&D systems, Minneapolis, MN) ELISA kits according to the manufacturer’s instructions.

### Splenocytes activation assay

Splenocytes isolated from CIA mice of indicated groups were incubated with anti-B220 (RA3-6B2) monoclonal antibody (mAb) to deplete B cells by immunomagnetic negative selection as describe previously^51^. The resulting cells were cultured (4 × 10^6^ cells/ml) with medium alone or CII (10 μg/ml) respectively for 72 hours. Total splenocytes from OVA-immunized mice of each group were stimulated with OVA (100 μg/ml) for 48 hours.

After incubation, for 96-well flat-bottom plates, [^3^H] thymidine assay was used to evaluate the cell proliferation. For 24-well plates, the supernatants were collected to determine levels of cytokines by ELISA.

### *In vitro* Tfh cell differentiation

Purification of naïve splenic CD4^+^ T cells (CD4^+^ CD44^–^CD62L^+^) from normal C57BL/6 mice was conducted using our previously reported methods^56^. Naïve CD4^+^ T cells were cultured with anti-CD3 mAb (5 μg/mL) and anti-CD28 mAb (2 μg/mL) for 4 days under different skewing conditions: Th0: anti-IFN-γ (10 μg/ml), anti-IL-4 (10 μg/ml, BD Bioscience); and Tfh: anti- IFN-γ (10 μg/ml), anti-IL-4 (10 μg/ml), IL-21 (50 ng/ml, Peprotech). SM934 (10 μM) was added to the culture simultaneously.

### Gene expression analysis

The total RNA was isolated from splenocytes of CIA mice treated with saline or SM934 (10 mg/kg) using RNAsimple total RNA kit (Tiangen Biotech, Beijing, China). and a one-step real-time PCR assay was performed with SYBR Green PCR Reagents (Qiagen, Valencia, CA, USA). Relative quantitation of mRNA expression of IL-21 and GAPDH was calculated using the ΔΔCt method. The primers sequence were as follows: for IL-21, 5′-GGA CCC TTG TCT GTC TGG TAG-3′ (forward) and 5′-TGT GGA GCT GAT AGA AGT TCA GG-3′ (reverse); and for GAPDH, 5′-GCC TCA AGG TAT TGC TGG AC-3′ (forward) and 5′-ACC TTG TTT GCC AGG TTC AC-3′ (reverse).

### Western blotting

B cell-depleted splenocytes from normal C57BL/6 mice (4 × 10^6^ cells/ml) were stimulated with/without IL-21 (10 ng/ml, Peprotech, Rocky Hill, NJ) and SM934 (10 μM) for 0 to 60 minutes.

Cell cultures or splenocytes isolated from CIA mice of individual group were directly lysed in sodium dodecyl sulfate (SDS) sample buffer containing protease inhibitor cocktail (Roche Life Science, Mannheim, Germany)^57^. Whole cell lysates were fractionated on 10% SDS-polyacrylamide gel electrophoresis (PAGE) and Western blotted with antibodies to phosphorylated STAT3 (Cell Signaling Technology, Beverly, MA) and GAPDH (KangChen Biotechnology, China). The densities of the bands were quantified with a computerized densitometer (Image J Launcher, Broken Symmetry Software).

### Statistical analysis

Statistical analysis was performed using GraphPad Prism 6.0 statistical software. Comparisons between 2 groups were performed using an unpaired 2-tailed *t*-test. For experiment involving multiple groups, one-way analysis of variance (ANOVA) followed by Turkey’s multiple comparison test was used, except for analysis of clinical scores which used two-way ANOVA followed by Dunnett’s multiple comparison test. *P* values less than 0.05 were considered significant.

## Additional Information

**How to cite this article**: Lin, Z.-M. *et al.* Artemisinin analogue SM934 attenuate collagen-induced arthritis by suppressing T follicular helper cells and T helper 17 cells. *Sci. Rep.* **6**, 38115; doi: 10.1038/srep38115 (2016).

**Publisher's note:** Springer Nature remains neutral with regard to jurisdictional claims in published maps and institutional affiliations.

## Supplementary Material

Supplementary Figures

Supplementary Information

## References

[b1] Li Y. Qinghaosu (artemisinin): chemistry and pharmacology. Acta pharmacologica Sinica 33, 1141–1146, doi: 10.1038/aps.2012.104 (2012). 22922345 PMC4003104

[b2] Paddon C. J. *et al.* High-level semi-synthetic production of the potent antimalarial artemisinin. Nature 496, 528–532, doi: 10.1038/nature12051 (2013). 23575629

[b3] Wang J. *et al.* Artemisinin directly targets malarial mitochondria through its specific mitochondrial activation. Plos one 5, e9582 (2010). 20221395 10.1371/journal.pone.0009582 PMC2833198

[b4] Wu W.-M. *et al.* Ferrous ion induced cleavage of the peroxy bond in qinghaosu and its derivatives and the DNA damage associated with this process. Chem. Commun. 2213–2214 (1996).

[b5] Wu W.-M. *et al.* Unified mechanistic framework for the Fe (II)-induced cleavage of qinghaosu and derivatives/analogues. The first spin-trapping evidence for the previously postulated secondary C-4 radical. Journal of the American Chemical Society 120, 3316–3325 (1998).

[b6] Zhou W. L. *et al.* A novel artemisinin derivative, 3-(12-beta-artemisininoxy) phenoxyl succinic acid (SM735), mediates immunosuppressive effects *in vitro* and *in vivo*. Acta pharmacologica Sinica 26, 1352–1358, doi: 10.1111/j.1745-7254.2005.00232.x (2005). 16225758

[b7] Wang J. X. *et al.* Investigation of the immunosuppressive activity of artemether on T-cell activation and proliferation. British journal of pharmacology 150, 652–661, doi: 10.1038/sj.bjp.0707137 (2007). 17262016 PMC2189761

[b8] Wang Z. *et al.* Anti-inflammatory properties and regulatory mechanism of a novel derivative of artemisinin in experimental autoimmune encephalomyelitis. Journal of immunology 179, 5958–5965 (2007). 10.4049/jimmunol.179.9.5958 17947669

[b9] Wang J. X. *et al.* Suppressive effect of a novel water-soluble artemisinin derivative SM905 on T cell activation and proliferation *in vitro* and *in vivo*. European journal of pharmacology 564, 211–218, doi: 10.1016/j.ejphar.2007.01.092 (2007). 17349993

[b10] Wang J. X. *et al.* The new water-soluble artemisinin derivative SM905 ameliorates collagen-induced arthritis by suppression of inflammatory and Th17 responses. British journal of pharmacology 153, 1303–1310, doi: 10.1038/bjp.2008.11 (2008). 18264129 PMC2275452

[b11] Li T. T. *et al.* Artemisinin analogue SM934 ameliorates the proteinuria and renal fibrosis in rat experimental membranous nephropathy. Acta pharmacologica Sinica 36, 188–199, doi: 10.1038/aps.2014.134 (2015). 25619396 PMC4326791

[b12] Hou L. F. *et al.* SM934 treated lupus-prone NZB x NZW F1 mice by enhancing macrophage interleukin-10 production and suppressing pathogenic T cell development. PLoS One 7, e32424, doi: 10.1371/journal.pone.0032424 (2012). 22389703 PMC3289663

[b13] Hou L. F. *et al.* Oral administration of artemisinin analog SM934 ameliorates lupus syndromes in MRL/lpr mice by inhibiting Th1 and Th17 cell responses. Arthritis and rheumatism 63, 2445–2455, doi: 10.1002/art.30392 (2011). 21484768

[b14] Li X. *et al.* Artemisinin analogue SM934 ameliorates murine experimental autoimmune encephalomyelitis through enhancing the expansion and functions of regulatory T cell. PLoS One 8, e74108, doi: 10.1371/journal.pone.0074108 (2013). 24009768 PMC3756992

[b15] Wu Y. *et al.* Therapeutic effects of the artemisinin analog SM934 on lupus-prone MRL/lpr mice via inhibition of TLR-triggered B-cell activation and plasma cell formation. Cellular & molecular immunology 13, 379–390, doi: 10.1038/cmi.2015.13 (2016). 25942599 PMC4856803

[b16] Victora G. D. & Nussenzweig M. C. Germinal centers. Annual review of immunology 30, 429–457, doi: 10.1146/annurev-immunol-020711-075032 (2012). 22224772

[b17] Jacob J., Kelsoe G., Rajewsky K. & Weiss U. Intraclonal generation of antibody mutants in germinal centres. Nature 354, 389–392, doi: 10.1038/354389a0 (1991). 1956400

[b18] Calame K. L. Plasma cells: finding new light at the end of B cell development. Nature immunology 2, 1103–1108 (2001). 11725300 10.1038/ni1201-1103

[b19] Crotty S. Follicular helper CD4 T cells (TFH). Annual review of immunology 29, 621–663, doi: 10.1146/annurev-immunol-031210-101400 (2011). 21314428

[b20] McHeyzer-Williams M., Okitsu S., Wang N. & McHeyzer-Williams L. Molecular programming of B cell memory. Nature reviews. Immunology 12, 24–34, doi: 10.1038/nri3128 (2012). PMC3947622 22158414

[b21] King C., Tangye S. G. & Mackay C. R. T follicular helper (TFH) cells in normal and dysregulated immune responses. Annual review of immunology 26, 741–766, doi: 10.1146/annurev.immunol.26.021607.090344 (2008). 18173374

[b22] Jiang S. H., Shen N. & Vinuesa C. G. Posttranscriptional T cell gene regulation to limit Tfh cells and autoimmunity. Current opinion in immunology 37, 21–27, doi: 10.1016/j.coi.2015.09.003 (2015). 26432764

[b23] Patakas A. *et al.* Th17 effector cells support B cell responses outside of germinal centres. PLoS One 7, e49715, doi: 10.1371/journal.pone.0049715 (2012). 23166752 PMC3500323

[b24] Mitsdoerffer M. *et al.* Proinflammatory T helper type 17 cells are effective B-cell helpers. Proceedings of the National Academy of Sciences of the United States of America 107, 14292–14297, doi: 10.1073/pnas.1009234107 (2010). 20660725 PMC2922571

[b25] Hsu H. C. *et al.* Interleukin 17-producing T helper cells and interleukin 17 orchestrate autoreactive germinal center development in autoimmune BXD2 mice. Nature immunology 9, 166–175, doi: 10.1038/ni1552 (2008). 18157131

[b26] Lei L., Zhong X. N., He Z. Y., Zhao C. & Sun X. J. IL-21 induction of CD4+ T cell differentiation into Th17 cells contributes to bleomycin-induced fibrosis in mice. Cell biology international 39, 388–399 (2015). 25492803 10.1002/cbin.10410

[b27] Wei L., Laurence A., Elias K. M. & O’Shea J. J. IL-21 is produced by Th17 cells and drives IL-17 production in a STAT3-dependent manner. Journal of Biological Chemistry 282, 34605–34610 (2007). 17884812 10.1074/jbc.M705100200 PMC2323680

[b28] Firestein G. S. Immunologic mechanisms in the pathogenesis of rheumatoid arthritis. Journal of clinical rheumatology: practical reports on rheumatic & musculoskeletal diseases 11, S39–44 (2005). 16357749 10.1097/01.rhu.0000166673.34461.33

[b29] Firestein G. S. Evolving concepts of rheumatoid arthritis. Nature 423, 356–361, doi: 10.1038/nature01661 (2003). 12748655

[b30] Liu R. *et al.* Allogeneic mesenchymal stem cells inhibited T follicular helper cell generation in rheumatoid arthritis. Sci Rep 5, 12777, doi: 10.1038/srep12777 (2015). 26259824 PMC4531289

[b31] Ma J. *et al.* Increased frequency of circulating follicular helper T cells in patients with rheumatoid arthritis. Clinical & developmental immunology 2012, 827480, doi: 10.1155/2012/827480 (2012). 22649468 PMC3357937

[b32] Simpson N. *et al.* Expansion of circulating T cells resembling follicular helper T cells is a fixed phenotype that identifies a subset of severe systemic lupus erythematosus. Arthritis & Rheumatism 62, 234–244 (2010). 20039395 10.1002/art.25032

[b33] Zhang X. *et al.* Identification of follicular helper T cells as a novel cell population potentially involved in the pathogenesis of Rheumatoid Arthritis (HUM3P. 251). The Journal of Immunology 194, 121.111-121.111 (2015).

[b34] Wang J. *et al.* High frequencies of activated B cells and T follicular helper cells are correlated with disease activity in patients with new-onset rheumatoid arthritis. Clinical & Experimental Immunology 174, 212–220 (2013). 23786438 10.1111/cei.12162 PMC3828824

[b35] Li G. *et al.* Interleukin-17A promotes rheumatoid arthritis synoviocytes migration and invasion under hypoxia by increasing MMP2 and MMP9 expression through NF-κB/HIF-1α pathway. Molecular immunology 53, 227–236 (2013). 22960198 10.1016/j.molimm.2012.08.018

[b36] Kato H., Endres J. & Fox D. A. The roles of IFN-γ versus IL-17 in pathogenic effects of human Th17 cells on synovial fibroblasts. Modern Rheumatology 23, 1140–1150 (2013). 23306426 10.1007/s10165-012-0811-x PMC3710715

[b37] Bajtner E., Nandakumar K. S., Engström Å. & Holmdahl R. Chronic development of collagen-induced arthritis is associated with arthritogenic antibodies against specific epitopes on type II collagen. Arthritis research & therapy 7, R1148 (2005). 16207332 10.1186/ar1800 PMC1257444

[b38] Stuart J. M. & Dixon F. J. Serum transfer of collagen-induced arthritis in mice. The Journal of experimental medicine 158, 378–392 (1983). 6886622 10.1084/jem.158.2.378 PMC2187334

[b39] Zotos D. *et al.* IL-21 regulates germinal center B cell differentiation and proliferation through a B cell–intrinsic mechanism. The Journal of experimental medicine 207, 365–378 (2010). 20142430 10.1084/jem.20091777 PMC2822601

[b40] Williams R. O. Collagen-induced arthritis in mice. Target Discovery and Validation Reviews and Protocols: Volume 2: Emerging Molecular Targets and Treatment Options 265–284 (2007).

[b41] Kannan K., Ortmann R. A. & Kimpel D. Animal models of rheumatoid arthritis and their relevance to human disease. Pathophysiology 12, 167–181 (2005). 16171986 10.1016/j.pathophys.2005.07.011

[b42] Luross J. A. & Williams N. A. The genetic and immunopathological processes underlying collagen-induced arthritis. Immunology 103, 407–416 (2001). 11529930 10.1046/j.1365-2567.2001.01267.x PMC1783255

[b43] Hu Y. L., Metz D. P., Chung J., Siu G. & Zhang M. B7RP-1 blockade ameliorates autoimmunity through regulation of follicular helper T cells. Journal of immunology 182, 1421–1428 (2009). 10.4049/jimmunol.182.3.1421 19155489

[b44] Quezada S. A., Jarvinen L. Z., Lind E. F. & Noelle R. J. CD40/CD154 interactions at the interface of tolerance and immunity. Annu. Rev. Immunol. 22, 307–328 (2004). 15032580 10.1146/annurev.immunol.22.012703.104533

[b45] Jordan M. B., Mills D. M., Kappler J., Marrack P. & Cambier J. C. Promotion of B cell immune responses via an alum-induced myeloid cell population. Science 304, 1808–1810 (2004). 15205534 10.1126/science.1089926

[b46] Bauquet A. T. *et al.* The costimulatory molecule ICOS regulates the expression of c-Maf and IL-21 in the development of follicular T helper cells and TH-17 cells. Nature immunology 10, 167–175 (2009). 19098919 10.1038/ni.1690 PMC2742982

[b47] Sun X. *et al.* Adiponectin exacerbates collagen-induced arthritis via enhancing Th17 response and prompting RANKL expression. Scientific reports 5 (2015). 10.1038/srep11296 PMC4462752 26063682

[b48] Ma C. S. *et al.* Functional STAT3 deficiency compromises the generation of human T follicular helper cells. Blood 119, 3997–4008, doi: 10.1182/blood-2011-11-392985 (2012). 22403255 PMC3355712

[b49] Wan C. K. *et al.* Opposing roles of STAT1 and STAT3 in IL-21 function in CD4+ T cells. Proceedings of the National Academy of Sciences of the United States of America 112, 9394–9399, doi: 10.1073/pnas.1511711112 (2015). 26170288 PMC4522759

[b50] Konforte D., Simard N. & Paige C. J. IL-21: an executor of B cell fate. Journal of immunology 182, 1781–1787, doi: 10.4049/jimmunol.0803009 (2009). 19201828

[b51] Zhou R. *et al.* (5R)-5-Hydroxytriptolide attenuated collagen-induced arthritis in DBA/1 mice via suppressing interferon-γ production and its related signaling. Journal of Pharmacology and Experimental Therapeutics 318, 35–44 (2006). 16574782 10.1124/jpet.106.101113

[b52] Fu Y.-F. *et al.* S-adenosyl-L-homocysteine hydrolase inactivation curtails ovalbumin-induced immune responses. Journal of Pharmacology and Experimental Therapeutics 316, 1229–1237 (2006). 16326921 10.1124/jpet.105.093369

[b53] Bhattacharya P. *et al.* A novel pancreatic β-cell targeting bispecific-antibody (BsAb) can prevent the development of Type 1 diabetes in NOD mice. Clinical immunology (Orlando, Fla.) 153, 187–198, doi: 10.1016/j.clim.2014.04.014 (2014). PMC4077286 24792135

[b54] Hollister K. *et al.* Insights into the role of Bcl6 in follicular Th cells using a new conditional mutant mouse model. The Journal of Immunology 191, 3705–3711 (2013). 23980208 10.4049/jimmunol.1300378 PMC3783642

[b55] Kawai R. *et al.* Evaluation of primary and secondary responses to a T-cell-dependent antigen, keyhole limpet hemocyanin, in rats. Journal of immunotoxicology 10, 40–48 (2013). 22953734 10.3109/1547691X.2012.691122

[b56] He S. J. *et al.* Therapeutic effects of DZ2002, a reversible SAHH inhibitor, on lupus-prone NZBxNZW F1 mice via interference with TLR-mediated APC response. Acta pharmacologica Sinica 35, 219–229, doi: 10.1038/aps.2013.167 (2014). 24374810 PMC4651227

[b57] Elshabrawy H. A. *et al.* Identification of a broad-spectrum antiviral small molecule against severe acute respiratory syndrome coronavirus and Ebola, Hendra, and Nipah viruses by using a novel high-throughput screening assay. Journal of virology 88, 4353–4365, doi: 10.1128/JVI.03050-13 (2014). 24501399 PMC3993759

