# Supplementary material for: Artemisinin analogue SM934 attenuate collagen-induced arthritis by suppressing T follicular helper cells and T helper 17 cells
Source: Sci Rep. 2016 Nov 29;6:38115. doi: 10.1038/srep38115 (PMC5126690; doi:10.1038/srep38115)
Supplement: Supplementary Figures [file srep38115-s1.pdf]

Title: Artemisinin analogue SM934 attenuate collagen-induced arthritis by suppressing T follicular helper cells and T helper 17 cells

Authors:

Ze-Min Lin [a,b] , Xiao-Qian Yang[b], Feng-Hua Zhu[b], Shi-Jun He[b] , Wei Tang[b] & Jian-Ping Zuo [a, b].

[a] Laboratory of Immunology and Virology, Shanghai University of Traditional Chinese Medicine, Shanghai 201203, People's Republic of China. [b]Laboratory of Immunopharmacology, Shanghai Institute of Materia Medica, Chinese Academy of Sciences, Shanghai 201203, People's Republic of China. Correspondence and requests for materials should be addressed to J.-P. Z. (email: jpzuo@simm.ac.cn) or W.T. (email: tangwei@simm.ac.cn) or S.-J.H. (email: heshijun@simm.ac.cn)

Supplementary Figure S1

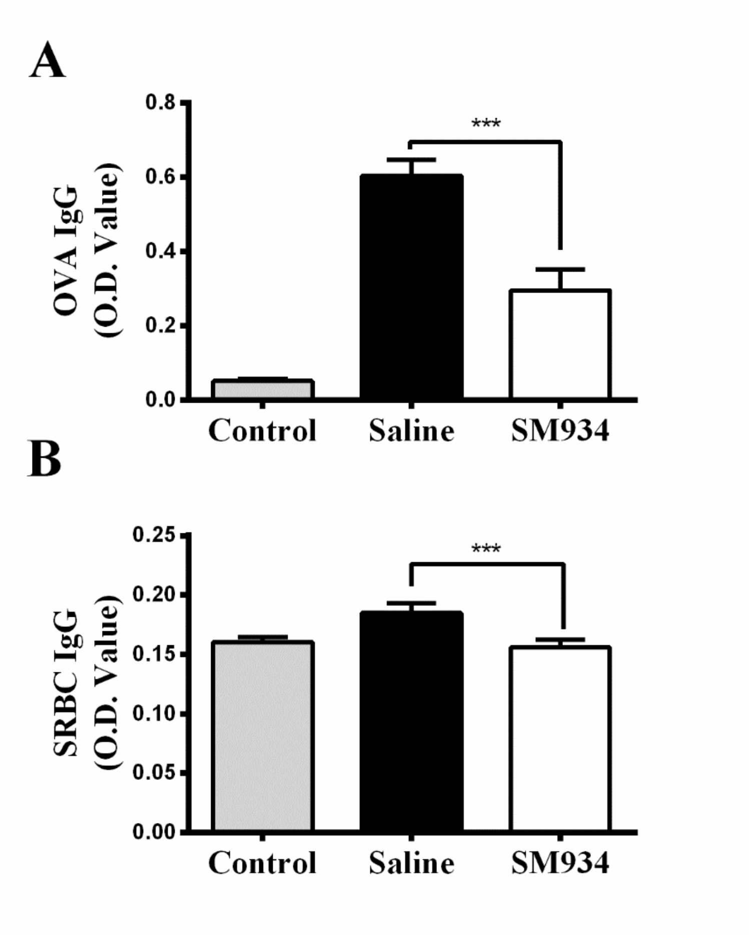

Supplementary Figure S1. SM934 decreased serum antigen-specific antibodies in SRBC- and OVA-immunized mice. (A) Serum levels of total anti-SRBC IgG of SRBC-immunized mice and normal control mice. (B) Serum levels of total anti-OVA IgG of OVA-immunized mice and normal control mice. Values are the mean ± SEM. ***P < 0.001 compared with the saline-treated SRBC- or OVA-immunized mice (saline). Results are derived from 2 independent experiments with similar pattern in each treated group (n=5 in each group).

Supplementary Figure S2

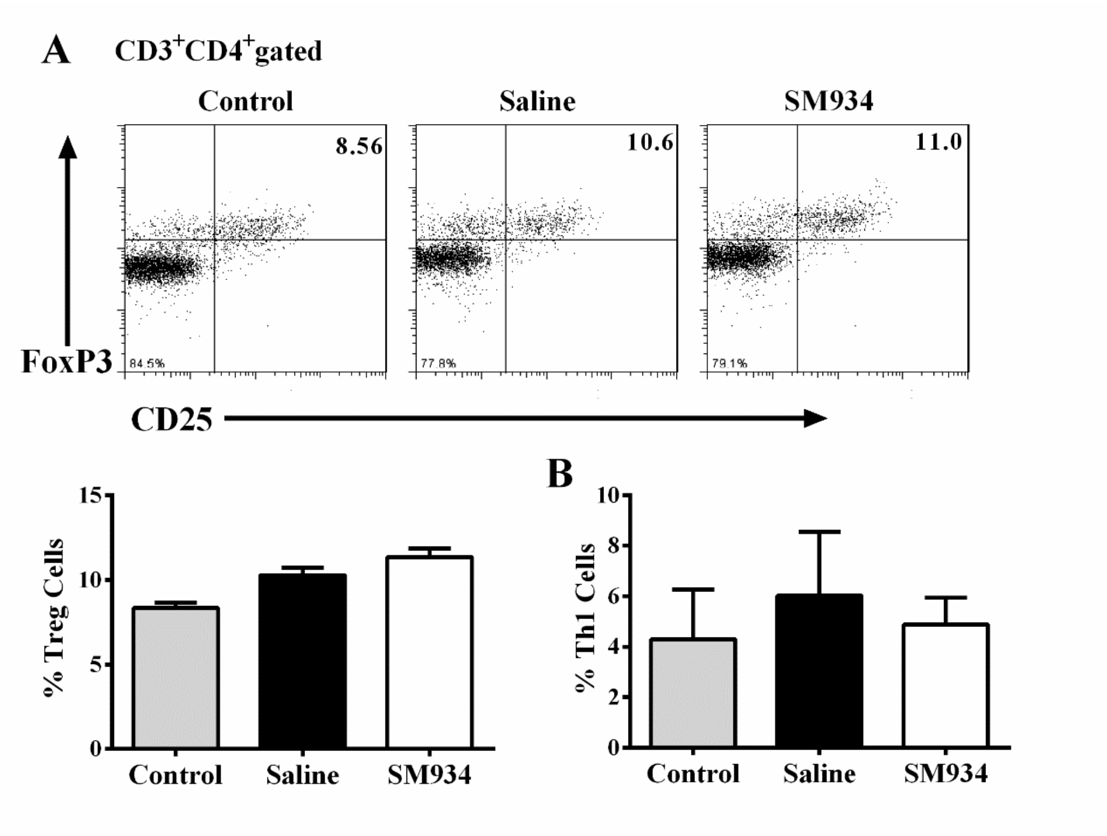

Supplementary Figure S2. Effect of SM934 on Th1 and Treg subsets in CIA mice. (A) Representative (up) and statistical results (low) of Treg cells percentage in splenocytes from CIA mice. (B) Statistical results of Th1 cells percentage in splenocytes from CIA mice. Values are the mean ± SEM. Results are derived from 2 independent experiments with similar pattern in each treated group (n=8 in each group).

Supplementary Figure S3

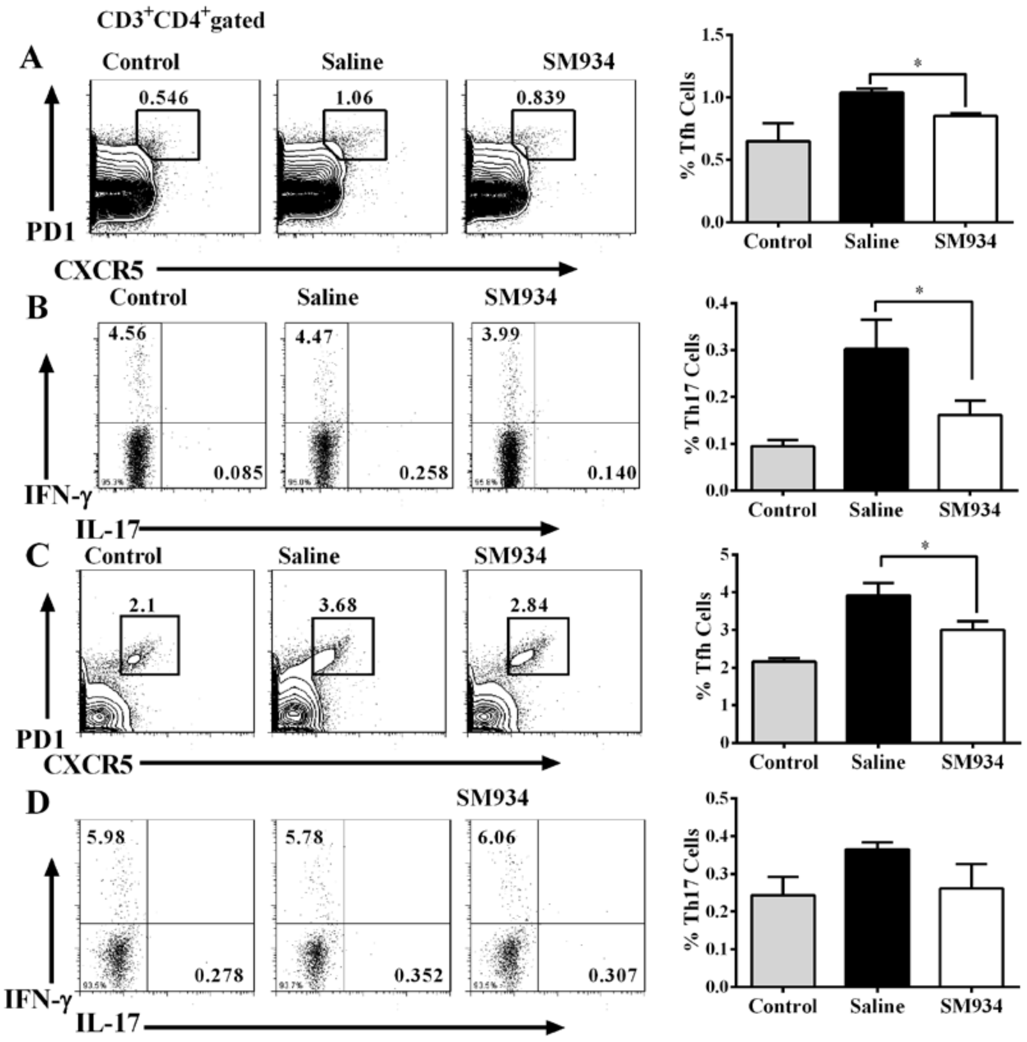

Supplementary Figure S3. SM934 abolished Tfh and Th17 population in SRBC- and OVA-immunized mice. Frequencies of Tfh cells and Th17 cells in splenocytes from SRBC-immunized mice (A and B) or OVA-immunized mice(C and D) were determined by flow cytometry, results were all showed on $CD3^{+}CD4^{+}$ T cell gate. The representative and statistical results were showed. Values are the mean ± SEM. *p < 0.05 compared with the saline-treated CIA mice (saline). Results are derived from 2 independent experiments with similar pattern in each treated group (n=5 in each group).
