# Supplementary material for: Artemisinin analogue SM934 attenuate collagen-induced arthritis by suppressing T follicular helper cells and T helper 17 cells
Source: Sci Rep. 2016 Nov 29;6:38115. doi: 10.1038/srep38115 (PMC5126690; doi:10.1038/srep38115)
Supplement: Supplementary Information [file srep38115-s2.pdf]

Title: Artemisinin analogue SM934 attenuate collagen-induced arthritis by suppressing T follicular helper cells and T helper 17 cells

Authors:

Ze-Min Lin [a,b] , Xiao-Qian Yang[b], Feng-Hua Zhu[b], Shi-Jun He[b] , Wei Tang[b] & Jian-Ping Zuo [a, b].

[a] Laboratory of Immunology and Virology, Shanghai University of Traditional Chinese Medicine, Shanghai 201203, People's Republic of China. [b]Laboratory of Immunopharmacology, Shanghai Institute of Materia Medica, Chinese Academy of Sciences, Shanghai 201203, People's Republic of China. Correspondence and requests for materials should be addressed to J.-P. Z. (email: jpzuo@simm.ac.cn) or W.T. (email: tangwei@simm.ac.cn) or S.-J.H. (email: heshijun@simm.ac.cn)

Full-length blots of Figure 6C:

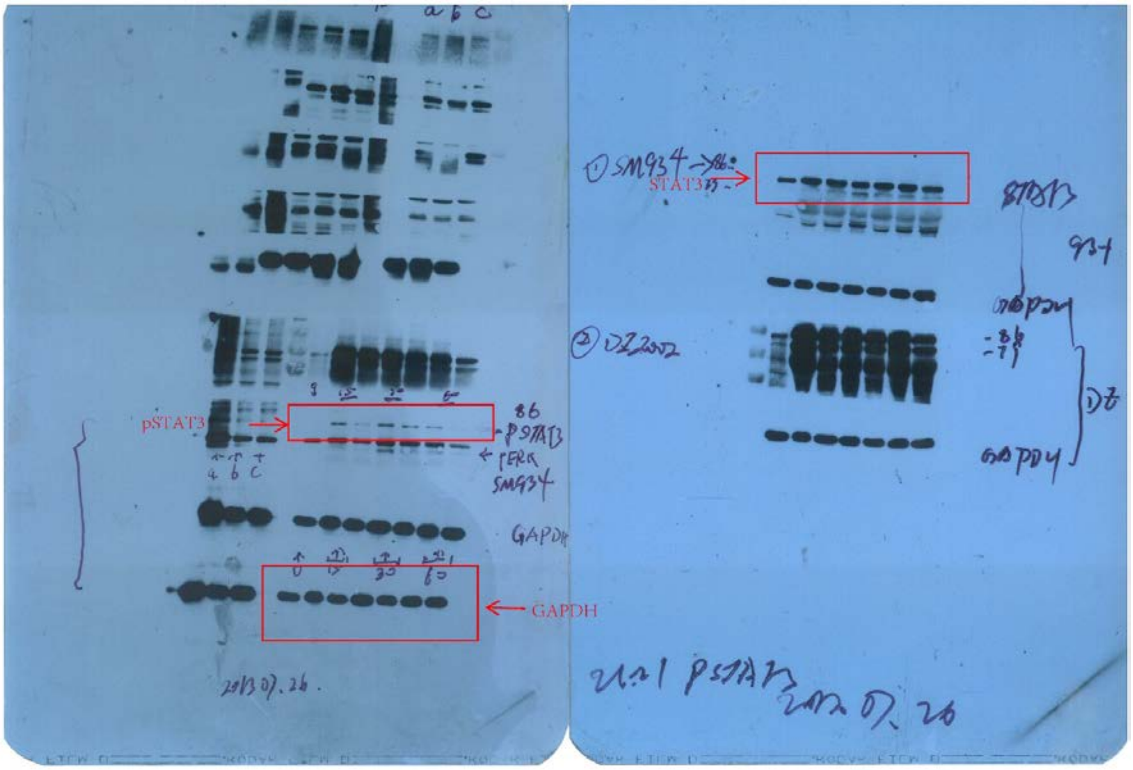

The boxes indicating the blots we used in Figure 6C, and arrows pointing to the specific proteins.
